# Differential Shedding and Antibody Kinetics of Zika and Chikungunya Viruses, Brazil

**DOI:** 10.3201/eid2502.180166

**Published:** 2019-02

**Authors:** Fernando A. Bozza, Andres Moreira-Soto, Alexandra Rockstroh, Carlo Fischer, Alessandra D. Nascimento, Andrea S. Calheiros, Christian Drosten, Patrícia T. Bozza, Thiago Moreno L. Souza, Sebastian Ulbert, Jan Felix Drexler

**Affiliations:** National Institute of Infectious Diseases Evandro Chagas, Oswaldo Cruz Foundation (FIOCRUZ), Rio de Janeiro, Brazil (F.A. Bozza, A.D. Nascimento);; D’Or Institute for Research and Education, Rio de Janeiro (F.A. Bozza, A.D. Nascimento);; Charité-Universitätsmedizin Berlin, corporate member of Freie Universität Berlin, Humboldt-Universität zu Berlin, and Berlin Institute of Health, Institute of Virology, Berlin, Germany (A. Moreira-Soto, C. Fischer, C. Drosten, J.F. Drexler);; Fraunhofer Institute for Cell Therapy and Immunology, Leipzig, Germany (A. Rockstroh, S. Ulbert);; Instituto Oswaldo Cruz, Rio de Janeiro (A.S. Calheiros, P.T. Bozza);; German Centre for Infection Research, Berlin (C. Drosten, J.F. Drexler);; Center for Technological Development in Health, Rio de Janeiro (T.M.L. Souza);; Martsinovsky Institute of Medical Parasitology, Tropical and Vector-Borne Diseases, Sechenov University, Moscow, Russia (J.F. Drexler)

**Keywords:** Zika virus, chikungunya virus, real time RT-PCR, serology, diagnosis, viruses, vector-borne infections, Brazil, arboviruses, flaviviruses

## Abstract

In seroconversion panels obtained from patients from Brazil, diagnostic testing for Zika virus infection was improved by combining multiple antibody isotypes, techniques, and antigens, but sensitivity remained suboptimal. In contrast, chikungunya virus diagnostic testing was unambiguous. Recurrent recent arbovirus infections suggested by serologic data and unspecific symptoms highlight the need for exhaustive virologic testing.

In 2013, Zika virus and chikungunya virus (CHIKV) emerged in Latin America (*1*,*2*). Their overlapping symptoms challenge accurate diagnosis on the basis of clinical manifestations (*3*). Direct Zika virus and CHIKV detection is limited to the acute phase of infection (*4*). Serologic detection of Zika virus–specific antibodies is hampered by low specificity and sensitivity of tests because of immune responses elicited by prior infection with other endemic flaviviruses (e.g., dengue virus [DENV]) (*5*,*6*). In addition, lack of adequate specimens limits studies evaluating the performance of diagnostic tests in tropical areas (*7*,*8*). To evaluate these challenges, we analyzed virus shedding and antibody responses over time in patients in Brazil sampled during the 2016 Zika virus and CHIKV outbreaks.

## The Study

**Figure 1:**
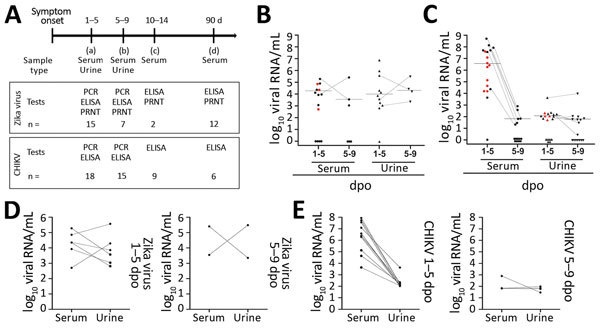
Overview of diagnostic testing and shedding dynamics for Zika virus and CHIKV among patients in Brazil, 2016. A) Timeline of sampling and number of samples for each test. B, C) Zika virus (B) and CHIKV (C) loads in different body fluids at 1–5 and 5–9 dpo. Black dots indicate single samples. Red dots indicate samples taken 1–5 dpo that were negative 5–9 dpo. Dotted lines indicate paired samples that were positive at both time points. Bold line indicates the median. D, E) Viral loads of Zika virus (D) and CHIKV (E) in paired urine and serum samples from individual patients, 1–5 and 5–9 dpo. Data were analyzed using GraphPad Prism 5 (GraphPad Software, Inc., https://www.graphpad.com). CHIKV, chikungunya virus; dpo, days post–symptom onset; PRNT, plaque reduction neutralization test.

We prospectively sampled patients in 4 time points up to 90 days post–symptom onset (dpo) (Table 1; Figure 1, panel A; Appendix). The cohort comprised 15 patients with acute Zika virus infection (5 male, 10 female; median age 39.0 years [interquartile range 31.0–44.0 years]) and 18 patients with acute CHIKV infection (10 male, 8 female; median age 39.0 years [interquartile range 31.0–57.3 years]), determined by detection of viral RNA in blood or urine 1–9 dpo (Appendix Figures 1, 2). All Zika virus belonged to the Asian lineage (*2*), and all CHIKV to the East/Central/South African lineage, according to envelope-based typing.

**Table 1:** Sampling details for retrospective study of differential shedding and antibody kinetics of Zika virus and CHIKV, Brazil, 2016*

Sample no.	Virus detected	Days from symptom onset to sampling	Collection date of acute-phase samples
DQ005	Zika virus	2	Mar 14
DQ028	Zika virus	1	Mar 21
DQ042	Zika virus	3	Mar 23
DQ47	Zika virus	2	Mar 28
DQ049	Zika virus	1	Mar 28
DQ058	Zika virus	4	Mar 30
DQ60	Zika virus	2	Mar 30
DQ62	Zika virus	3	Mar 30
DQ75	Zika virus	3	Apr 4
DQ77	Zika virus	5	Apr 5
DQ108	Zika virus	2	Apr 13
DQ116	Zika virus	2	Apr 14
DQ125	Zika virus	3	Apr 18
DQ131	Zika virus	5	Apr 18
DQ246	Zika virus	5	Jun 24
DQ030	CHIKV	3	Mar 21
DQ045	CHIKV	5	Mar 24
DQ054	CHIKV	2	Mar 30
DQ056	CHIKV	2	Mar 30
DQ057	CHIKV	3	Mar 30
DQ061	CHIKV	2	Mar 30
DQ071	CHIKV	4	Apr 4
DQ074	CHIKV	1	Apr 4
DQ079	CHIKV	3	Apr 5
DQ083	CHIKV	3	Apr 6
DQ085	CHIKV	4	Apr 7
DQ097	CHIKV	3	Apr 11
DQ113	CHIKV	5	Apr 13
DQ144	CHIKV	4	Apr 25
DQ170	CHIKV	2	May 3
DQ195	CHIKV	2	May 11
DQ210	CHIKV	2	May 16
DQ220	CHIKV	4	May 17

*CHIKV, chikungunya virus.

At enrollment, Zika virus patients most frequently reported fever, rash, and arthralgia (80% each), and CHIKV patients most frequently reported arthralgia (100%), fever (89%), and myalgia (89%) (Table 2). No co-infection with Zika virus, CHIKV, or DENV was detected by real-time reverse transcription PCR (rRT-PCR). However, serologic analyses found that 4 (27%) Zika virus–infected patients also had CHIKV IgM at enrollment, and 1 (7%) had DENV IgM (Appendix Table 1, Figure 3). Similarly, 3 (17%) CHIKV-infected patients had Zika virus IgM, and 4 (22%) CHIKV-infected patients had DENV IgM at enrollment (Appendix Figure 4). We cannot exclude the possibility of cross-reactivity between Zika virus–specific and DENV-specific antibodies because 2 CHIKV patients simultaneously showed Zika virus and DENV IgM in an envelope-based ELISA (Appendix Table 2). Seventy-nine percent of Zika virus and 83% of CHIKV patients showed serologic evidence for past DENV infection at enrollment (Appendix Figures 1, 2). Thus, recent infections with heterologous arboviruses might bias attributing infection-specific symptoms for Zika virus and CHIKV.

**Table 2:** Symptoms of Zika virus and CHIKV reported by patients at enrollment 1–5 days after symptom onset, Brazil, 2016*

Symptom	Zika virus, no. (%), n = 15	CHIKV, no. (%), n = 18
Rash	12 (80)	9 (50)
Fever	12 (80)	16 (89)
Arthralgia	12 (80)	18 (100)
Myalgia	9 (60)	16 (89)
Cephalea	8 (53)	12 (67)
Retro-orbital pain	5 (33)	8 (44)
Edema	4 (27)	3 (17)
Nausea, vomiting	3 (20)	6 (33)
Conjunctivitis	2 (13)	5 (28)

*CHIKV, chikungunya virus.

Consistent with previous studies (*4*,*9*), Zika virus loads in serum and urine were low up to 9 dpo (≈10^4^ RNA copies/mL) (Figure 1, panel B), whereas CHIKV loads were high ≈100-fold higher (≈10^6^ RNA copies/mL) (Figure 1, panel C). However, unlike with Zika virus, CHIKV loads decreased significantly (p<0.001 by *t* test) from 5 dpo onward, and viral loads in urine were consistently low (Figure 1, panels D, E).

**Figure 2:**
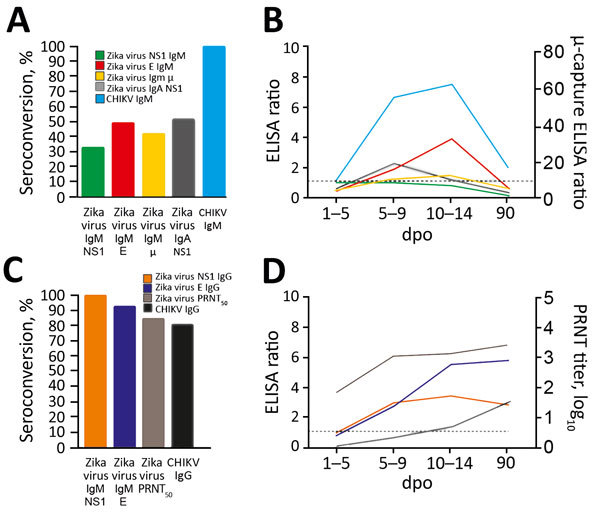
Zika virus and CHIKV antibody dynamics among samples from patients in Brazil, 2016. A) Percentage seroconversion for markers of acute infection with Zika virus and CHIKV (IgM NS1–based Zika virus ELISA, IgM envelope-based Zika virus ELISA, IgM μ-capture Zika virus ELISA, IgA NS1-based Zika virus ELISA, IgM CHIKV ELISA) at any time point. B) Median ELISA ratios for Zika virus and CHIKV IgM and IgA over time. C) Percentage seroconversion for markers of convalescence after Zika virus and CHIKV infection (IgG NS1-based Zika virus ELISA and IgG envelope-based Zika virus ELISA, Zika virus PRNT_50_, IgG CHIKV ELISA) at any time point. D) Median ELISA ratios for Zika virus and CHIKV IgG over time. Numbers of specimens per time point are shown in Figure 1. Dashed lines indicate signal-to-cutoff ratios of >1.1 considered positive for all ELISAs except μ-capture ELISA, for which the dashed line indicates a signal-to-cutoff ratio of >10, considered positive by the manufacturer. See Appendix Figure 5 for the percentage de novo seroconversion of Zika virus and CHIKV in different assays per time point. CHIKV, chikungunya virus; dpo, days post–symptom onset; E, envelope; NS, nonstructural protein; PRNT, plaque reduction neutralization test.

Next, to assess the antibody kinetics of Zika virus and CHIKV, we measured antibody responses over time by commercial and in-house serologic tests. In a widely used nonstructural (NS) protein 1 antigen-based ELISA, Zika virus IgM seroconversion was low (33% [5/15]), whereas CHIKV IgM seroconversion was 100% using an envelope-based ELISA (p<0.0001 by Fisher exact test) (Figure 2, panel A; Appendix Tables 1, 2). Use of an in-house envelope-based ELISA increased the Zika virus IgM detection rate to 50% (7/14), and use of a commercially available μ-capture ELISA increased it to 43% (6/14) (Figure 2, panel A). Despite differential sensitivity, concordant results from different assays suggest comparable specificity of IgM detection (Appendix Table 1). The use of NS1-based IgA as a marker of acute infection increased the detection rate to 53% (8/15) over that of the NS1-based IgM ELISA. All IgM-positive patients also showed IgA, which increased during acute and subacute phases of infection and decreased during convalescence (Figure 2, panel B; Appendix Figure 3). This finding supports the usability of IgA-based serologic methods as an alternative or additional marker to IgM-based methods to detect acute Zika virus infection. The detection rate increased 2-fold when we used NS1-based IgA from when we used NS1-based IgM 5–9 dpo, suggesting that IgA could be used at later stages of infection (Appendix Figures 1, 5). Our findings indicate that serologic detection of acute Zika virus infection can be improved ≈2-fold by use of different antibody classes and antigens but remains poorly sensitive in flavivirus-endemic areas.

All Zika virus–infected patients showed IgG responses across the 4 time points in >1 assay (Figure 2, panels C, D). Plaque reduction neutralization tests (PRNTs) were negative for 2 of 14 rRT-PCR–confirmed Zika virus cases detected by NS1-based IgG ELISA. Without rRT-PCR confirmation, these cases would have been classified false positive (Appendix Table 1). This observation might be explained by differential sensitivity of PRNT and ELISA (*10*) or false-positive results of the Zika virus NS1-based ELISA in secondary flavivirus infections (*6*). Similarly, the antibody kinetics of Zika virus NS1-based IgG, envelope-based IgG, and PRNT suggested either relatively early IgG seroconversion or cross-reactivity during acute stages of infection resulting from unspecific immune responses against other flaviviruses (*11*) (Figure 2, panel D). In contrast, CHIKV IgG seroconversion occurred at later stages (Figure 2, panel D; Appendix Figure 5), possibly associated with strong and long-lasting CHIKV-specific IgM responses (Appendix Figure 4).

## Conclusions

We provide pivotal data on Zika virus and CHIKV diagnostic challenges in a Latin American setting. Limitations of our study include the relatively small number of patients, sampling at heterogeneous dpo and heterogeneous numbers of samples per dpo, and lack of acutely DENV-infected patients to assess test specificity. The strengths of our study include rRT-PCR–confirmed infections, waiving the need to define serologic assays prone to cross-reactivity as standards, sampling during Zika virus and CHIKV outbreaks (*1*,*2*), sequential sampling of patients up to 90 dpo, use of multiple antigens and immunoglobulin classes, and the combination of molecular and serologic testing methods.

Our data suggest reliable diagnostic testing for acute CHIKV infections by IgM detection from 5 dpo onward. This finding might enable waiving labor-intense and costly molecular protocols in many patients, minimizing costs for public health systems and cohort studies investigating arbovirus pathogenesis. However, reliability of CHIKV serologic diagnostic tests must be reevaluated for co-circulating genotypes (*12*) and for the antigenically related Mayaro virus (*13*) if it emerges in Latin America.

The difficulties of adequately diagnosing Zika virus infections in areas to which it is endemic have major implications for public health. Reliable testing for flaviviruses in such areas will be key for epidemiologic studies on Zika virus and assessments of the safety of flavivirus vaccination programs, as illustrated by more severe dengue infections in DENV-seronegative individuals who received a live attenuated dengue vaccine (*14*).

For pregnant women and couples intending pregnancy, accurate diagnosis of acute or past Zika virus infection is crucial. The steep increase in requests for abortion in Latin America illustrates the effect of the Zika virus outbreak on reproductive medicine (*15*). 

Our results highlight that definite exclusion of acute Zika virus infections is challenging in a considerable proportion of patients. However, although limited by a small number of samples, our data highlight the attainability of more accurate Zika virus diagnostic testing by combining molecular and serologic tests using different antibody classes, antigens, and methods and by monitoring an increase of IgG titers in follow-up serum samples. Our data will help clinicians and health authorities build reliable diagnostic algorithms for Zika virus and CHIKV and highlight that exhaustive testing of arboviral infections is required for attributing frequencies of infection-specific symptoms.

Appendix Additional information on differential shedding and antibody kinetics of Zika and chikungunya viruses, Brazil.
