# Supplementary material for: Differential Shedding and Antibody Kinetics of Zika and Chikungunya Viruses, Brazil
Source: Emerg Infect Dis. 2019 Feb;25(2):311–5. doi: 10.3201/eid2502.180166 (PMC6346451; doi:10.3201/eid2502.180166)
Supplement: Appendix — Additional information on differential shedding and antibody kinetics of Zika and chikungunya viruses, Brazil. [file 18-0166-Techapp-s1.pdf]

Article DOI: https://doi.org/10.3201/eid2502.180166

Differential Shedding and Antibody Kinetics of Zika Virus and Chikungunya Virus, Brazil

Appendix

Appendix Table 1. Serologic data for 14 Zika virus patients, Brazil, 2016*

		Zika virus							CHIVK		DENV				
Patient	Age, y	**IgA NS1**	**IgM NS1**	**IgM E**	**IgM μ**	**IgG NS1**	**IgG E**	**PRNT 50**	**IgM RE**	**IgG RE**	**IgA FV**	**IgM FV**	**IgM E**	**IgG FV**	**IgG E**
DQ005	32	+	–	–	+	+	+	+	–	–	–	–	+	+	+
DQ028	44	+	+	NA	NA	+	NA	NA	–	–	–	–	NA	+	NA
DQ042	35	+	+	+	+	+	+	+	+	–	–	–	+	+	–
DQ47	36	+	+	+	+	+	+	+	–	–	–	+	–	+	–
DQ049	40	+	+	–	+	+	+	+	–	–	–	–	–	+	+
DQ058	39	–	–	–	–	+	+	+	–	–	–	–	–	+	+
DQ60	60	+	–	+	+	+	+	+	–	–	–	–	+	+	+
DQ62	44	+	–	+	–	+	+	+	–	–	–	–	+	+	+
DQ75	30	–	–	+	–	+	+	+	–	–	–	–	–	+	–
DQ77	28	–	–	–	–	+	+	–	+	+	+	–	–	+	+
DQ108	21	–	–	–	–	+	–	–	–	–	–	–	–	+	+
DQ116	46	–	–	–	–	+	+	+	–	–	–	–	–	+	+
DQ125	41	–	–	+	–	+	+	+	+	+	+	+	+	+	+
DQ131	31	+	+	+	+	+	+	+	–	–	–	+	–	+	–
DQ246	NA	–	–	–	–	+	+	+	+	+	+	–	–	+	+
Total		8	5	7	6	15	13	12	4	3	3	3	5	15	10

*ELISA or PRNT results at any time point of sampling. CHIKV, chikungunya virus; DENV, dengue virus; E, envelope; FV, full virus; NS, nonstructural protein; NA, samples not available; PRNT, plaque-reduction neutralization test; RE, recombinant; +, positive; –, negative.

Appendix Table 2. Serologic data for 18 CHIKV patients, Brazil, 2016*

		Zika virus						CHIKV		DENV			
Patient	Age, y	**IgA NS1**	**IgM NS1**	**IgM E**	**IgM μ**	**IgG NS1**	**IgG E**	**IgM RE**	**IgG RE**	**IgM FV**	**IgM E**	**IgG FV**	**IgG E**
DQ030	45	+	–	–	–	+	+	+	–	–	–	+	+
DQ045	27	–	–	–	–	–	–	+	+	–	–	–	–
DQ054	57	–	–	–	–	+	+	+	+	+	–	+	+
DQ056	41	–	–	+	–	–	–	+	–	–	+	–	–
DQ057	38	–	–	–	–	+	–	+	+	–	–	+	+
DQ061	35	–	–	–	–	+	–	+	+	–	+	+	+
DQ071	69	–	–	–	–	–	–	+	+	–	–	+	+
DQ074	33	–	–	–	–	+	+	+	+	–	–	+	+
DQ079	33	–	–	–	–	+	+	+	+	–	–	+	+
DQ083	58	+	–	–	–	+	+	+	+	–	+	+	+
DQ085	40	–	–	–	–	+	+	+	+	+	–	+	–
DQ097	29	–	–	–	–	–	–	+	+	–	–	+	+
DQ113	42	–	–	–	–	+	+	+	+	–	–	+	+
DQ144	31	–	–	–	–	+	+	+	+	–	–	+	+
DQ170	24	–	–	+	–	+	+	+	+	–	+	+	+
DQ195	31	–	–	–	–	–	–	+	–	–	–	+	+
DQ210	75	–	–	–	–	–	–	+	+	–	–	+	+
DQ220	85	–	–	–	–	+	+	+	–	–	–	+	+
Total positive		2	0	2	0	12	10	18	15	2	4	16	15

*ELISA results at any time point of sampling. CHIKV, chikungunya virus; DENV, dengue virus; E, envelope; FV, full virus; NS, nonstructural protein; RE, recombinant; +, positive; –, negative.

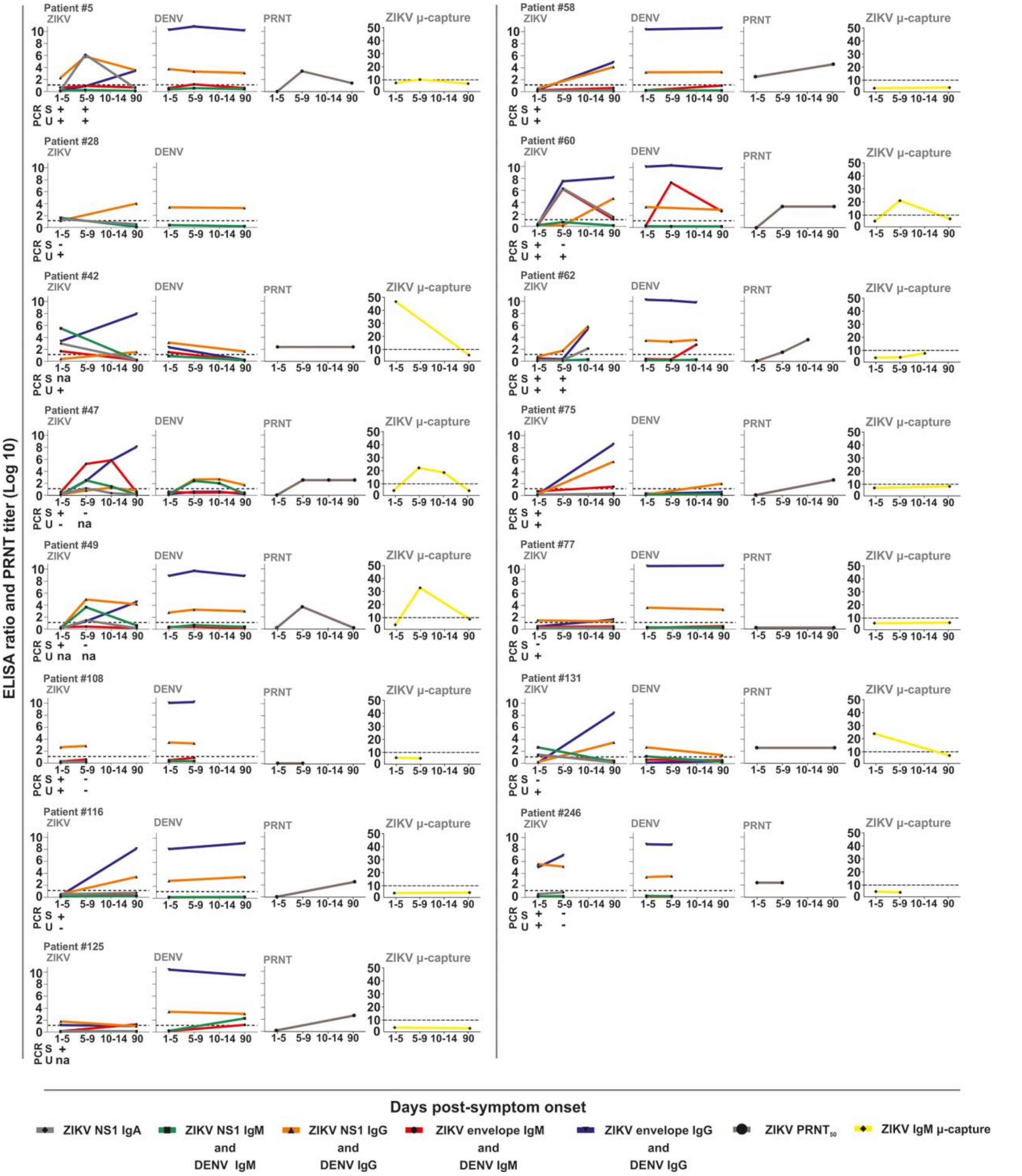

Appendix Figure 1. Antibody kinetics and real-time reverse transcription PCR results for ZIKV and DENV of 14 Zika patients. Below each panel, PCR results for serum and urine are shown. DENV, dengue virus; NA, sample not available; NS, nonstructural protein; PRNT, plaque-reduction neutralization test; ZIKV, Zika virus.

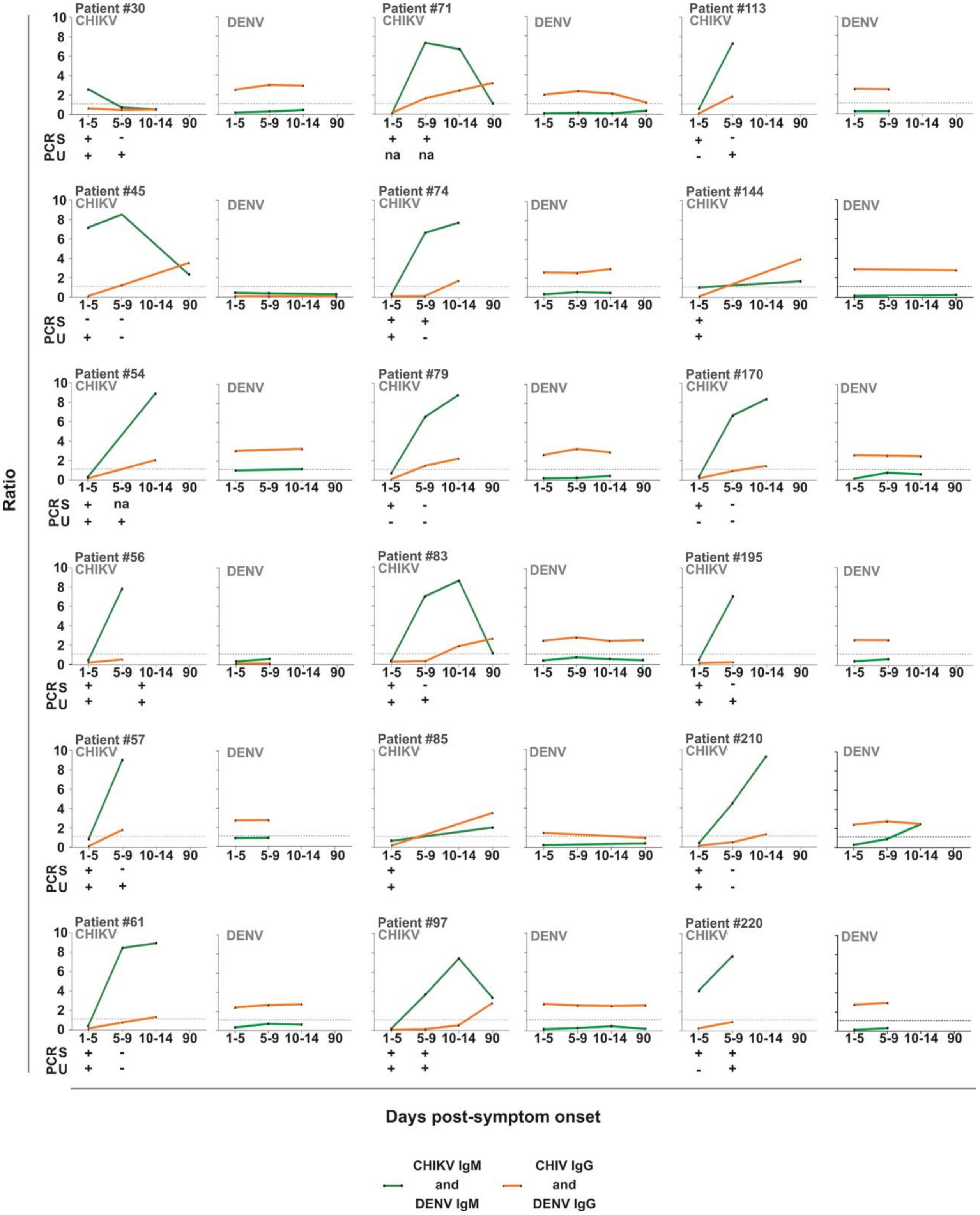

Appendix Figure 2. Antibody kinetics and real-time reverse transcription PCR results for CHIKV and DENV of 18 chikungunya patients, Brazil, 2016. Below each panel, PCR results for serum (S) and urine (U) are shown. CHIKV, chikungunya virus; DENV, dengue virus.

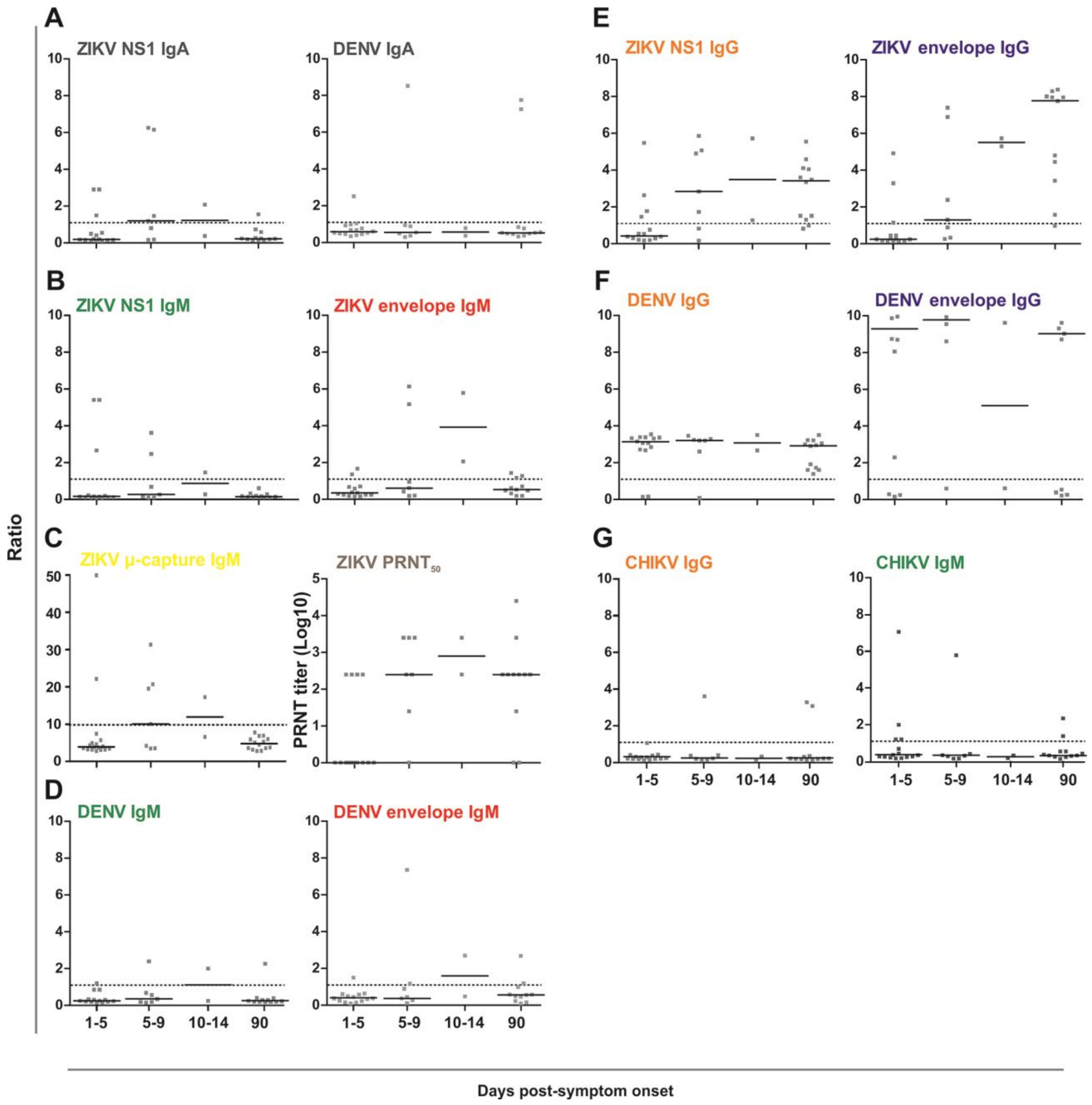

Appendix Figure 3. ELISA ratios and plaque-reduction neutralization test titers of 14 Zika virus–positive patients, Brazil, 2016. Lines show the median. Dashed lines indicate signal-to-cutoff ratios of ≥1.1 considered positive. For the µ-capture ELISA, the dashed line indicates a signal-to-cutoff ratio of ≥10. CHIKV, chikungunya virus; DENV, dengue virus; NS, nonstructural protein; PRNT, plaque-reduction neutralization test; ZIKV, Zika virus

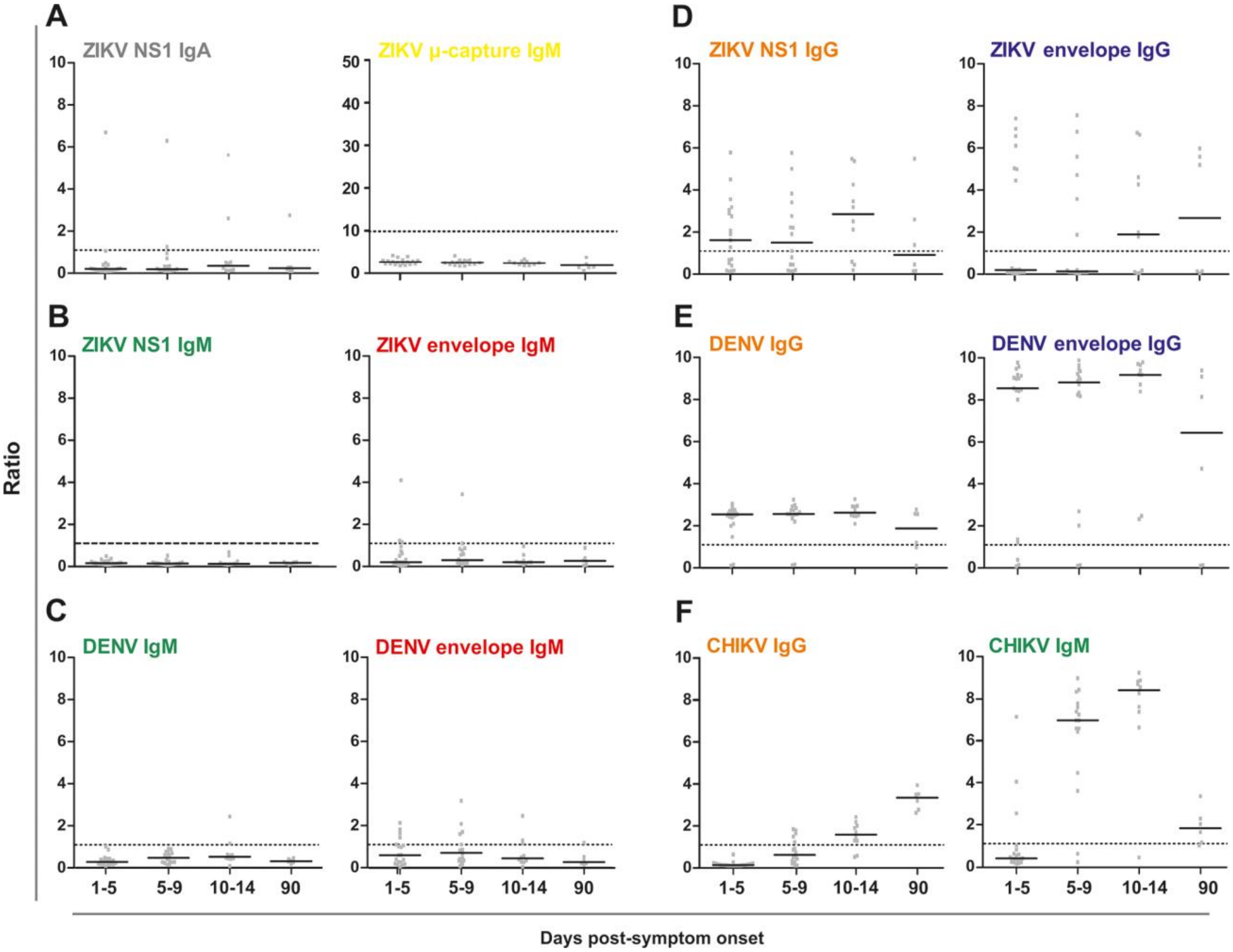

Appendix Figure 4. ELISA ratios for 18 CHIKV-positive patients, Brazil, 2016. Dashed lines indicate signal-to-cutoff ratios of >1.1 considered positive. For the μ-capture ELISA, dashed line indicates a signal-to-cutoff ratio of >10. CHIKV, chikungunya virus; DENV, dengue virus; NS, nonstructural protein; ZIKV, Zika virus.

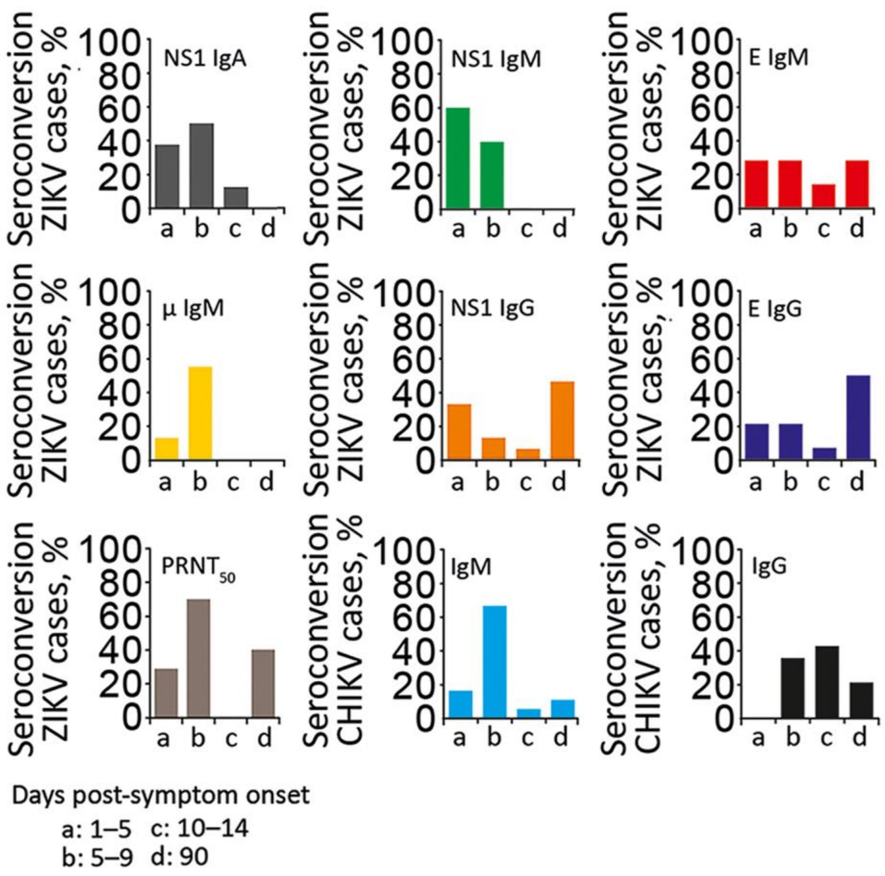

Appendix Figure 5. Percentage de novo seroconversion of Zika virus and CHIKV in different assays per time point. Total numbers of patients that seroconverted for Zika virus per assay, antigen, and antibody were as follows: NS1 IgA (n = 8), NS1 IgM (n = 5), envelope IgM (n = 7), NS1 IgG (n = 15), envelope IgG (n = 14) μ-capture IgM (n = 6), and PRNT50 (n = 12). Total numbers of specimens that seroconverted for CHIKV were as follows: IgM (n = 18), IgG (n = 14). NS, nonstructural protein; PRNT, plaque-reduction neutralization test.
